# The developmental trajectory of ^1^H-MRS brain metabolites from childhood to adulthood

**DOI:** 10.1093/cercor/bhae046

**Published:** 2024-03-01

**Authors:** Alice R Thomson, Hannah Hwa, Duanghathai Pasanta, Benjamin Hopwood, Helen J Powell, Ross Lawrence, Zeus G Tabuenca, Tomoki Arichi, Richard A E Edden, Xiaoqian Chai, Nicolaas A Puts

**Affiliations:** Department of Forensic and Neurodevelopmental Sciences, Institute of Psychiatry, Psychology & Neuroscience, King’s College London, 16 De Crespigny Park, London, SE5 8AF, United Kingdom; MRC Centre for Neurodevelopmental Disorders, Department of Neuro­developmental Disorders, New Hunt's House, Guy's Campus, King's College London, London, SE1 1UL, United Kingdom; Department of Forensic and Neurodevelopmental Sciences, Institute of Psychiatry, Psychology & Neuroscience, King’s College London, 16 De Crespigny Park, London, SE5 8AF, United Kingdom; Department of Forensic and Neurodevelopmental Sciences, Institute of Psychiatry, Psychology & Neuroscience, King’s College London, 16 De Crespigny Park, London, SE5 8AF, United Kingdom; Department of Forensic and Neurodevelopmental Sciences, Institute of Psychiatry, Psychology & Neuroscience, King’s College London, 16 De Crespigny Park, London, SE5 8AF, United Kingdom; Department of Forensic and Neurodevelopmental Sciences, Institute of Psychiatry, Psychology & Neuroscience, King’s College London, 16 De Crespigny Park, London, SE5 8AF, United Kingdom; Division of Cognitive Neurology, Department of Neurology, Johns Hopkins University, 1629 Thames Street Suite 350, Baltimore, MD 21231, United States; Department of Statistical Methods, University of Zaragoza, Pedro Cerbuna 12, Zaragoza, 50009, Spain; MRC Centre for Neurodevelopmental Disorders, Department of Neuro­developmental Disorders, New Hunt's House, Guy's Campus, King's College London, London, SE1 1UL, United Kingdom; Centre for the Developing Brain, Department of Perinatal Imaging & Health, 1st Floor, South Wing, St Thomas’ Hospital, London, SE1 7EH, United Kingdom; Russell H. Morgan Department of Radiology and Radiological Science, The Johns Hopkins University School of Medicine, 601 North Caroline Street, Baltimore, MD 21287, United States; F.M. Kirby Research Centre for Functional Brain Imaging, Kennedy Krieger Institute, 707 North Broadway, Baltimore, MD 21205, United States; Department of Neurology and Neurosurgery, McGill University, QC H3A2B4, Canada; Department of Forensic and Neurodevelopmental Sciences, Institute of Psychiatry, Psychology & Neuroscience, King’s College London, 16 De Crespigny Park, London, SE5 8AF, United Kingdom; MRC Centre for Neurodevelopmental Disorders, Department of Neuro­developmental Disorders, New Hunt's House, Guy's Campus, King's College London, London, SE1 1UL, United Kingdom

**Keywords:** edited MRS, lifespan, metabolites, MRS, neurodevelopment

## Abstract

Human brain development is ongoing throughout childhood, with for example, myelination of nerve fibers and refinement of synaptic connections continuing until early adulthood. *1H-Magnetic Resonance Spectroscopy* (^1^H-MRS) can be used to quantify the concentrations of endogenous metabolites (e.g. glutamate and γ -aminobutyric acid (GABA)) in the human brain in vivo and so can provide valuable, tractable insight into the biochemical processes that support postnatal neurodevelopment. This can feasibly provide new insight into and aid the management of neurodevelopmental disorders by providing chemical markers of atypical development. This study aims to characterize the normative developmental trajectory of various brain metabolites, as measured by ^1^H-MRS from a midline posterior parietal voxel. We find significant non-linear trajectories for GABA+ (GABA plus macromolecules), Glx (glutamate + glutamine), total choline (tCho) and total creatine (tCr) concentrations. Glx and GABA+ concentrations steeply decrease across childhood, with more stable trajectories across early adulthood. tCr and tCho concentrations increase from childhood to early adulthood. Total N-acetyl aspartate (tNAA) and Myo-Inositol (mI) concentrations are relatively stable across development. Trajectories likely reflect fundamental neurodevelopmental processes (including local circuit refinement) which occur from childhood to early adulthood and can be associated with cognitive development; we find GABA+ concentrations significantly positively correlate with recognition memory scores.

## Introduction

There is increasing interest in developing measures of neurotypical brain development to understand the underlying biological processes and provide a reference for assessing atypical developmental trajectories. Magnetic resonance imaging (MRI) and histological studies have provided evidence of extensive postnatal human brain development. For example, there is a widespread increase in cerebral white matter volume (Yakovlev and Lecours 1967; Jernigan et al. 1991; Giedd et al. 1999; Lebel et al. 2008; Jernigan et al. 2011; Bethlehem et al. 2022) and the fractional anisotropy of major white matter tracts from birth to early adulthood, indicative of post-natal nerve fiber myelination and alignment (Kinney et al. 1988; Giedd et al. 1999; Beaulieu 2002; Lebel et al. 2008; Coll et al. 2020). Concurrent with this is the refinement of local brain circuitry, as the initially excessive excitatory synaptic connections undergo activity dependent pruning (Changeux and Danchin 1976; Lebel et al. 2008; Selemon 2013). Accordingly, human cortical synaptic density peaks in early childhood (2–4 years of age), before decreasing until early adulthood (Huttenlocher 1979), consistent with works in non-human primates (Rakic et al. 1986; Zecevic and Rakic 1991; Bourgeois and Rakic 1993). Increasing rates of cerebral glucose metabolism coincide with these developmental alterations in brain architecture (Chugani et al. 1987), as does behavioral and cognitive development e.g. language, working, motor skills and autobiographical memory (Kwon et al. 2002; Hampson et al. 2006; Demaster and Ghetti 2013; Ghetti and Bunge 2012; Chang et al. 2016; Lee et al. 2016; Breukelaar et al. 2018; Given-Wilson et al. 2018; O’Rawe et al. 2019).

As the vast majority of work has focused on the aforementioned structural changes, much less is known about the accompanying developmental changes in various brain metabolites and neurotransmission. *^1^H-Magnetic Resonance Spectroscopy* (^1^H-MRS) is a non-invasive MRI technique that can be used to quantify the concentrations of endogenous metabolites in the human brain in vivo (Puts and Edden 2012). ^1^H-MRS can thus provide valuable insights into the biochemical processes which likely underlie structural brain development. Furthermore, understanding developmental changes in neurochemistry can feasibly provide much needed chemical biomarkers of atypical neurodevelopment, which crucially, are likely to be more therapeutically tractable than existing structural indices (Blüml et al. 2013; Bethlehem et al. 2022).

^1^H-MRS commonly focuses on the brain’s high-concentration metabolites; total N-acetyl aspartate (tNAA), Myo-Inositol (mI), total choline (tCho), and total creatine (tCr). These molecules can be found in both neurons and glial cells (Rae 2014). tNAA MRS signal is composed N-acetylaspartylglutamate (NAAG) and, in majority, its precursor N-acetyl aspartate (NAA; Frahm et al. 1991). Both are highly concentrated in neurons, where NAA is synthesized by N-acetyltransferase 8-like (NAT8L) in the mitochondria and plays key roles in mitochondrial metabolic pathways (Patel and Clark 1979; Rae 2014; Siddiqui et al. 2016). Choline is generally found within the choline-containing compounds phosphocholine, phosphatidylcholine, and glycerophosphocholine, which, along with free choline, make up the composite MRS signal referred to as tCho (Miller et al. 1996; Rae 2014). These molecules are essential for cell membrane integrity and signaling, as well as acetylcholine synthesis within cholinergic neurons and they are found in myelin sheath (Rae 2014). The ^1^H-MRS tCr signal is composed of creatine and its phosphorylated form phosphocreatine, formed when ATP reacts with creatine (a reaction catalyzed by creatine kinase). This reaction is reversible and can more readily release ATP compared to oxidative phosphorylation, meaning phosphocreatine acts as a fast energy store (Wallimann et al. 1992; Rae 2014). Understanding the maturational trajectory of these metabolites can thus give insights into the neurochemical integrity of the developing brain with more mechanistic detail compared to structural imaging alone.

In addition, specifically tailored “editing” ^1^H-MRS sequences can be used to estimate the concentrations of the principle inhibitory and excitatory neurotransmitters of the adult human brain, γ -aminobutyric acid (GABA) and glutamate (Glu), along with their precursor glutamine (Gln; Puts and Edden 2012; Mullins et al. 2014; Harris et al. 2017). Note that due to contamination with macromolecules which are also affected by the GABA-editing MRS sequences, GABA signal is denoted as GABA+ (GABA + macromolecules; Mullins et al. 2014). Similarly, due to the poor chemical shift dispersion achieved at 1.5–3 tesla field strengths, it is often not possible to distinguish Glu signal from that of its precursor Gln. Glx therefore describes the combined signals of Gln and Glu (Mullins et al. 2014). GABA, Glu, and Gln, have fundamental roles in regulating neuronal connectivity, synaptic neurotransmission, neural metabolism, and myelination (Madhavarao et al. 2005; Ghisleni et al. 2015; Serrano-Regal et al. 2020). During development, GABA and Glu are not “inhibitory” or “excitatory” per se, with GABA signaling known to evoke depolarisation of the neuronal membrane (excitation) early in postnatal development in many species including mammals (Ben-Ari et al. 1989; Dzhala et al. 2005; Kirmse et al. 2015).

Previous attempts to characterize the neurodevelopmental dynamics of these key brain metabolites have considered narrow, adult only (≥18) or child only (<18) cohorts (Chang et al. 1996; Saunders et al. 1999; Pfefferbaun et al. 1999; Angelie et al. 2001; Kaiser et al. 2005; Sailasuta et al. 2008; Reyngoudt et al. 2012; Marsman et al. 2013; Gao et al. 2013; Blüml et al. 2013; Hädel et al. 2013; Silveri et al. 2013; Ding et al. 2016; Eylers et al. 2016; Puts et al. 2017; Schmitz et al. 2018; Lind et al. 2020; Saleh et al. 2020; Hupfeld et al. 2021), and/or have focused on very few metabolites (Ghisleni et al. 2015; Volk et al. 2019; Shimizu et al. 2017). Combined with small sample sizes, variation in brain region, and ^1^H-MRS quantification methods, this has led to highly conflicting outcomes. As such, deciphering individual metabolite developmental trajectories that are concurrent with developmental structural changes remains a challenge (Porges et al. 2021). Furthermore, most studies to date have used linear modeling approaches for metabolite trajectories, which may fail to capture subtle differences across development and thus do not truly reflect the rich and complex changes that likely occur during maturation (Lebel and Beaulieu 2011; Porges et al. 2021).

Accordingly, here we aim to characterize the developmental trajectory of six brain metabolites (GABA+, Glx, tNAA, tCho, mI and tCr), as measured by edited ^1^H-MRS from a posterior parietal cortex voxel (PPC) in a typically developing sample (86 participants) that spans structural and functional development (5–35 years old). As the PPC is associated with episodic memory formation and retrieval (Wagner et al. 2005; Vincent et al. 2006; Cabeza et al. 2008; Ciaramelli et al. 2008; Rugg and King 2018; Neri et al. 2021), we also aim to explore whether neurodevelopmental differences in PPC ^1^H-MRS trajectories are associated with development of cognitive function, by observing if GABA+/Glx concentrations in particular associate with recognition memory scores.

## Materials and methods

### Participants

A total of 117 participants participated in in vivo MRI imaging and cognitive testing. All participants consented to participation in the study through local Kennedy Krieger Institute and Johns Hopkins University IRB procedures. Children assented and their parents consented to participation. All participants were native English speakers, right-handed, had normal or corrected-to-normal vision, with no history of psychiatric, neurological, or developmental disorders. All participants had IQ > 85. Participant demographic data were collected at the time of scan. Thirty-one MRS dataset were excluded due to poor data quality (see quality control section below). As such, our analysis uses data from 86 participants between 5 and 35 years of age (40 females, 46 males, mean age = 16.45 years, SD = 7.24). The range of participant IQ scores was 85–138 (median IQ = 118). Median household income of participants was > $100 k (range: <$35 K—>$100 K).

### Memory task

Participants completed a source memory encoding task in the MRI after the MRS scan and were given a memory test after the MRI session. Encoding stimuli consisted of four blocks of 40 color images of commonly known, visually distinct objects overlaid on top of one of two backgrounds (beach or forest). Object images fell under one of seven categories (animal, clothing, fruit, vegetable, toy, tool, instrument). For each trial, participants answered either one of the two questions: “Do you like this object or dislike/not care about it?”, indicated by a smiling cartoon face and a neutral cartoon face, or “Is this a living or not living object”, indicated by a leaf and a leaf with a red “X” through it. The background and encoding questions were randomly assigned to each object, ensuring that each category had an equal distribution of the four background and question combinations. Each image was shown for 3 s (s), followed by a fixation screen, consisting of a white “+” symbol on a black background, for 1 to 9 s.

The memory test consisted of three blocks of 80 images either from the encoding task or new images from the same categories, totaling 240 images (80 new, 160 previously seen during the encoding task). Images were displayed in pseudorandom order, with no more than three consecutive images from either the new image set or from the encoding activity. For each image, participants were asked to first determine whether they remembered seeing the object during the encoding activity and also remember specific details (e.g. what the image looked like on the screen, what they were thinking at the time, etc.), did not remember seeing the object, or thought the object was familiar but could not recall additional details (denoted by the options “Remember,” “New,” and “Familiar” respectively; Gardiner 1988). If the participant chose either remember or familiar, they were then asked to answer which of the two backgrounds the object was shown with, followed by which of the two question prompts (living/non-living, or like/do not like) the object was shown with. The test was self-paced in which the next question was presented as soon as the participant pressed a response key.

The trials were categorized based on the answers provided during the testing task into “Hits” (old objects correctly identified as remember or familiar), “Miss” (old objects identified as “New”), “False Alarm” (new objects falsely identified as remember or familiar), and “Correct Rejection” (new objects identified as “New”). Hits were further divided into remember and familiar based upon the participants’ answer. Remember and familiar trials were further divided into those with correct source information (background image correct, encoding question correct, or both sources correct). Recognition memory accuracy was calculated by subtracting the percentage of false alarms from the percentage of hits (%Hits–%FA).

### **Magnetic resonance imaging/**  ^1^H-Magnetic resonance spectroscopy

MRI and ^1^H-MRS was performed on the Philips 3 Tesla Achieva scanner (Best, NL) at the F.M. Kirby Research Centre for Functional Brain Imaging at the Kennedy Krieger institute in Baltimore, USA. For all scans, a 32-channel head coil was used for receiver, and the body coil for transmit. Prior to MRS, a high-resolution (1 mm^3^ isotropic) T1-weighted MP-RAGE anatomical image was acquired for voxel placement and tissue segmentation.

**Fig. 1:**
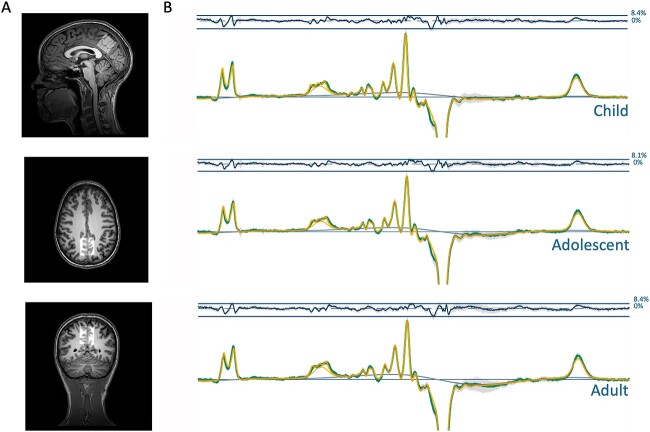
(A) Example voxel placement in posterior parietal cortex (PPC) (B) mean difference spectra (edit-ON—Edit-OFF) per age group (green), with example model fit (yellow) and model baseline (dark gray), median residual fits (gray error bars), and a ribbon plot representing the standard deviation across all spectra (top).

MRS data were acquired from a 27 mL voxel (3 × 3 × 3 cm^3^) placed over the PPC, centered on the midline (see Fig. 1A). MRS was performed using GABA+ selective MEshcher-Garwood Point RESolved Spectroscopy (MEGA-PRESS; Mescher et al. 1998) with the following parameters: 320 transients (160 ON and 160 OFF), 2048 data points, TE/TR 68/2000 ms with editing pulses placed at 1.9 ppm in the edit-ON acquisitions and 7.46 in the edit-OFF acquisitions and VAPOR water suppression. An interleaved unsuppressed water reference (Edden et al. 2016) with same parameters for water supressed scans was used (16 averages) to mitigate scanner drift and for subsequent eddy-current and phase corrections, and metabolite quantification.

### Data processing

Raw MRS data (both difference and edit-OFF spectra) were processed using Osprey (Version 2.4.0; Oeltzschner et al. 2020), an automated software for MRS analysis based in Matlab (version 2022a). We used the following Osprey processing steps: first, raw data were eddy current-corrected using the water reference followed by frequency and phase correction using robust spectral registration (Near et al. 2015; Mikkelsen et al. 2020; Oeltzschner et al. 2020), Fourier transformation and water signal removal. Edit-ON and edit-OFF spectra averages were subtracted to resolve GABA+ (at 3.02 ppm) from the overlaying metabolites (creatine, phosphocreatine, tNAA) which are subtracted out in the difference spectrum (Fig. 1B). Average metabolite spectra were modeled using a TE-specific simulated basis set and a flexible spline baseline available on Osprey and based on MRS vendor, pulse duration and scan sequence parameters (generated by fast spatially resolved 2D density-matrix simulation in the MATLAB toolbox FID-A; Simpson et al. 2017; Oeltzschner et al. 2020). Basis sets for macromolecule and lipid contributions were integrated as gaussian basis functions (Oeltzschner et al. 2020). Difference and edit-OFF spectra were modeled between 0.5 ppm and 4 ppm with linear baseline correction and a knot spacing of 0.55 ppm according to the Osprey model algorithm (Oeltzschner et al. 2020). Modeling was performed for 19 metabolites (ascorbic acid, aspartic acid, total Creatine, creatine methylene, GABA+, glycerophosphocholine, glutathione, glutamine, glutamate, myo-inositol, lactate, total N-acetylaspartate, n-acetylaspartylglutamate, total choline, phosphocholine, phosphocreatine, phosphatidylethanolamine, scyllo-inositol, taurine), five macromolecules and three lipids (MM09, MM12, MM14, MM17, MM20, Lip09, Lip13, Lip20) for both the edit-OFF and difference spectra (see supplementary materials for all raw data).

Here, we focus on GABA+ and Glx as well as tNAA, tCr, mI and tCho. While we report Glx, Osprey uses fitting to additionally produce Glu and Gln only estimates, whereby it is assumed that the majority of Glx is indeed Glu. These are reported in the supplementary but are not interpreted due to the inherent limitations of these estimates without individual metabolite quality metrics. Metabolite concentrations were estimated relative to the unsuppressed water signal in institutional units (i.u) and as a ratio relative to total creatine (e.g. tCho/tCr). We focus interpretation on estimated metabolite concentrations (i.u) due to the significant age effects observed for creatine concentrations within our study period. GABA+ was quantified in the difference spectra, while tNAA, tCho, tCr, mI, Glx (Glu and Gln) were quantified in the edit-OFF spectra. The Osprey co-registration module (via SPM version 12) was used to register MRS to the T1-weighted images acquired at the scan and segment the voxel volume into gray matter fraction (fGM), white matter fraction (fWM) and cerebrospinal fluid fraction (fCSF). Outputs were visually inspected to ensure accurate localization of the MRS voxel. Segmented T1 images were used to obtain tissue composition corrected water-scaled estimates of metabolite concentrations (i.u), whereby concentrations are scaled according to the assumption that metabolite concentrations in CSF are negligible (Gasparovic et al. 2006; Harris et al. 2015). Metabolite T1 and T2 relaxation effects were also accounted for (tissue water and metabolite; Gasparovic et al. 2006, Puts et al. 2013, Oeltzschner et al. 2020). Finally, “alpha correction” of GABA+ concentrations was performed in Osprey, with the assumption that GABA+ concentration is two times greater in GM compared to WM (Jensen et al. 2005; Harris et al. 2015), GABA+ concentrations were calculated using a group average voxel tissue composition (Harris et al. 2015).

### Spectral artifacts and quality control

Spectra were visually inspected by an experienced MRS data user (NP) blind to participant age. MRS spectra with significant artifacts due to motion and/or scanner drift and/or out of voxel echo and so indistinguishable GABA+ peaks at 3.02 ppm were excluded. Quantitative quality metrics (QM; signal to noise ratio (SNR), full width half maximum (FWHM), metabolite fit residuals and residual water amplitude) were then assessed visually through box plots, and a systematic inspection that involved isolating metrics that deviated three times the interquartile range above the third quartile or below the first quartile. Spectra of newly identified QM outliers were consulted to confirm whether the data was of poor quality. Overall, 21 datasets were excluded: 11 adult (25% of adult datasets), 2 adolescent (8% of adolescent datasets) and 8 child datasets (20% of child datasets). Box plot inspection of metabolite values ensured no extremely significant QM outliers remained. Data (*n* = 86) are reported based on consensus guidelines (Lin et al. 2021). QM were added as co-variates to statistical models.

### Statistical analysis

Statistical analysis of data was performed on RStudio (2 July 2022). We are primarily interested in the developmental trajectory of metabolites. However, as many existing studies focus on specific age groups (childhood (5–12), adolescence (13–18) and adulthood (18+)), we were also keen to establish differences in metabolite concentrations between these age groups to aid interpretation with respect to the literature. As such, data were analyzed both across all participants with age as a continuous variable, and categorically by age group (Table 1). Data are reported as mean (standard deviation) unless stated otherwise.

**Table 1:** Participant demographics by age group.

Age group	N (females/males)	Mean age (SD)	Mean IQ (SD)
Child (5 ≥ age < 12)	31 (15/16)	9.86 (1.66)	112.03 (13.26)
Adolescent (12 ≥ age < 18)	22 (10/12)	14.44 (1.48)	112.82 (11.48)
Adult (age ≥ 18)	33 (15/18)	29.98 (5.63)	119.88 (9.61)
All participants	86 (40/46)	16.45 (7.24)	115.24 (11.96)
*P* value			0.0157^*^

Note: *P* value indicates significant demographic difference between age groups (ANCOVA). *P*-values indicate significance of ANCOVA’s testing the main effect of age group on IQ, ^*^P < 0.05.

*Group comparisons* Data were first tested for normality using the Wilks-Shapiro test and tested for equal variance (of categorical groups) using Levene’s test, confirming that parametric tests were suitable. Analysis of covariance tests (ANCOVA) were used to determine the impact of age group on MRS QM, demographic information, and voxel tissue fractions. ANCOVAs were also used to determine the impact of age group on metabolite concentrations, with IQ, sex, fit residuals (edit-OFF or difference), SNR, FWHM (creatine or water) and voxel fGM included as covariates. Tukey’s honest significant difference (HSD) was used for post-hoc testing, accounting for multiple comparisons and unequal group sizes. Sex had no significant effect for any metabolite (excluding tCho) and thus males and females were pooled for further analysis. For tCho, sex effects were explored using Wilcoxon Signed Rank Tests with Bonferroni multiple comparison correction. Pearson correlation coefficients (using the *cor* function in R) were calculated to determine the association between metabolite concentration and age for each age group (children, adolescents, and adults).

*Modeling metabolite trajectories* Both linear and non-linear regression was used to estimate metabolite trajectories. For linear regressions we used the *lm* function in R, with metabolite as outcome and age as the predictor. Sex, IQ, SNR, FWHM (water or creatine), fGM and fit residuals (edit-OFF or difference) were added as predictors to the linear model. The likelihood ratio test was used to check this full model against nested models to confirm appropriateness. For tCho, exploratory linear regressions were performed for sex-separated data due to significant sex interactions.

For non-linear models we use Generalized Additive Models (GAM; Hastie 1992) using the “*gam”* function from the *mgcv* package in R (Wood 2011). GAM is a generalized additive modeling approach whereby the impact of the predictor variables on the dependent variable is modeled through a set number of flexible spline functions which can be linear or non-linear. Each spline making up the GAM has its own unique coefficient and splines are added together to produce the final model, from which we obtain several inferential parameters (R^2^, F statistic and *P* values). Neurometabolite concentrations were modeled using smooth (Gaussian process) functions of age, IQ, SNR, FWHM (water or creatine), fGM and fit residuals (difference or edit-OFF), while sex was added as a categorical predictor. The Restricted Maximum Likelihood method (REML) was used to select the Gaussian process smoothing parameters for each predictor. *“gam.check”* function (Wood 2011) was used to select a suitable number of smooth functions for each predictor (residuals should be randomly distributed). For GAM, the effective degrees of freedom (edf) of each smooth function (for each predictor) are assessed, these represent the complexity of the smoothing. Edf’s of 1 indicate a linear relationship between the dependant variable and predictor, while edfs of 2 indicate a quadratic relationship; greater edf values as such indicate a greater degree of non-linearity. Reference degrees of freedom and the F statistic were used to assess the significance of predictor smooth functions (significance meaning greater certainty of the shape of the smooth). Given the sex effects, sex-specific trajectories were plotted for tCho.

*Correlational analysis* In order to observe the interaction between the individual metabolites across development, we calculated Pearson correlation coefficients for every metabolite pair before plotting onto a correlation matrix. We did this per age group to assess whether metabolite cross-correlations qualitatively changed with age. Correlation coefficients were also calculated between Glx/GABA+ concentrations and recognition memory scores. The linear regression (*recognition memory ~ age + GABA+/Glx)* was used to control for age effects*.*

## Results

Participant demographics are reported in Table 1**.** Age group had a significant main effect on IQ (*P* < 0.05), with IQ significantly greater in adults compared to children and adolescent age groups.

In line with consensus reporting standards (MRSinMRS; Lin et al. 2021, MRS-Q; Peek et al. 2020), we report data QM to assess the quality of the MRS data and fit (Table 2). QM measures across subgroups met standard metrics confirming high quality spectra in the retained data (Mikkelsen et al. 2017).

**Table 2:** Quality of MRS data by age group.

**QM**	**Child**	**Adolescent**	**Adult**	**All**	***P* value**
**SNR (water)**	217.78 (27.53)	195.98 (34.99)	169.42 (30.57)	191.73 (53.62)	4.6e-07^*^^*^^*^^*^
**FWHM (water)**	4.26 (0.34)	4.56 (0.49)	5.05 (0.56)	4.63 (0.74)	6.51e-12^*^^*^^*^^*^
**Fit residuals (difference)**	3.56 (1.01)	2.92 (1.32)	2.57 (0.80)	2.98 (1.06)	0.000672 ^*^^*^^*^
**Fit residuals (OFF)**	16.10 (4.29)	11.70 (5.24)	8.49 (2.88)	11.73 (6.83)	1.13e-09^*^^*^^*^^*^
**Frequency shift (Hz)**	−2.42 (0.44)	−2.38 (0.86)	−2.39 (0.82)	−2.39 (0.78)	0.853
**GM fraction**	0.70 (0.061)	0.65 (0.040)	0.61 (0.041)	0.64 (0.073)	<2e-16 ^*^^*^^*^^*^
**WM fraction**	0.24 (0.052)	0.27 (0.039)	0.30 (0.046)	0.27 (0.059)	1.07e-08 ^*^^*^^*^^*^
**CSF fraction**	0.059 (0.029)	0.072 (0.031)	0.085 (0.040)	0.073 (0.035)	2.46e-05 ^*^^*^^*^

Note: Median (interquartile range (IQR)) are shown. Quality metrics include signal to noise ratio (SNR), full-width half maximum (FWHM), fit residuals (edit-OFF and difference spectra) and frequency shift (Hertz (Hz)). Gray matter (GM), white matter (WM) and cerebral spinal fluid (CSF) voxel fractions are also shown. *P*-values indicate significance of ANCOVA’s testing the main effect of age group on QM/tissue fractions. ^*^P < 0.05, ^*^^*^P < 0.01, ^*^^*^^*^P < 0.001, ^*^^*^^*^^*^P < 0.0001.

**Fig. 2:**
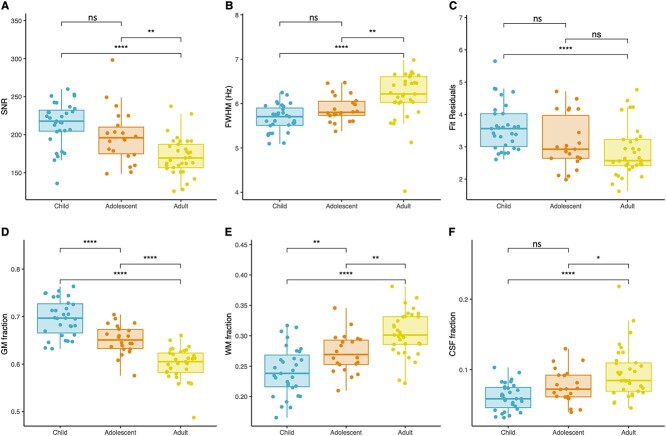
Quality metrics per age group. Following ANCOVA, Tukey HSD post-hoc correction was used to isolate significant differences in QM between the age groups for (A) SNR (B) FWHM (C) fit residuals (difference) (D) voxel GM fraction (E) voxel WM fraction (F) voxel CSF fraction. Ns: Non-significant, ^*^P < 0.05, ^*^^*^P < 0.01, ^*^^*^^*^P < 0.001, ^*^^*^^*^^*^P < 0.0001.

Age group differences were observed for SNR, FWHM and spectra fit residuals (edit-OFF and difference). Tukey post hoc analysis shown in Fig. 2 found that SNR of adult data was significantly lower compared to child (171.68 (25.06) versus 214.63 (29.05); *P* < 0.0001) and adolescent data (171.68 (25.06) versus 197.98 (34.50); *P* < 0.005), however SNR is still considered to be high for all age groups (Wilson et al. 2019). The adult data had significantly greater FWHM than child (5.08 (0.43) versus 4.52 (0.42); *P* < 0.0001) and adolescent data (5.08 (0.43) versus 4.27 (0.32); *P* < 0.005), however, FWHM is still considered to be small for each age group and represents a good shim (Wilson et al. 2019). Finally, edit-OFF and difference spectra fit residuals of child data were significantly greater than that of adult data (16.10 versus 11.70; *P* < 0.0001 and 3.62 versus 2.85; *P* < 0.001). We explore this in the discussion below.

### Tissue fractions

Age group showed a significant main effect on voxel tissue fractions (GM, WM, CSF; Table 2). Voxel GM fraction significantly decreased from children to adolescents to adults (Fig. 2), and negatively correlated with age (r = −0.78, *P* < 0.0001). Voxel WM and CSF fractions significantly increased between age groups from children to adolescents to adults (Fig. 2), positively correlating with age (r = 0.65, *P* < 0.001 and r = 0.42, *P* < 0.0001 respectively). Note that data presented below as estimated metabolite concentrations in i.u. is corrected for differences in voxel tissue composition.

### Neurochemistry per age group

Mean estimated metabolite concentrations (i.u) and metabolite creatine ratios (/tCr) are reported for each age group in Supplementary Table 1 (*n* = 86; 31 children, 22 adolescents and 33 adults). Following ANCOVA’s, age group showed a significant main effect on estimated concentrations of tCr, tCho and Glx (Supplementary Table 2). For creatine-ratio metabolite data, age group showed a significant main effect on tNAA/tCr, tCho/tCr, Glx/tCr, mI/tCr and GABA+/tCr (Supplementary Table 2).

Note residuals of fit, SNR, FWHM, fGM, sex and IQ were included as covariates in ANCOVA tests. Edit-off spectra fit residuals had a significant main effect on estimated tCho (F_2,83_ = 15.71, *P* < 0.05) and Glx (F_2,83_ = 96.32, *P* < 0.001; Supplementary Table 2). SNR had a significant main effect on estimated tCr (F_2,83_ = 12.58, *P* < 0.001), tCho (F_2,83_ = 7.60, *P* < 0.01) and mI (F_2,83_ = 9.53, *P* < 0.01). FWHM had a significant main effect on estimated tCr (F_2,83_ = 13.23, *P* = 0.00) and tNAA (F_2,83_ = 7.876, *P* < 0.001). fGM had a significant main effect on tCho (F_2,83_ = 4.49, *P* < 0.05). Sex had a significant main effect on estimated tCho (F_2,83_ = 9.183, *P* < 0.0001). Adult males had significantly greater tCho concentrations compared to adult females (2.35 (0.17) versus 2.60 (0.32); *P* < 0.05), with no significant differences in tCho concentrations between sexes in child or adolescent age groups (Supplementary Table 4). Similar results were observed for metabolite creatine ratio data (Supplementary Tables 2-4).

**Fig. 3:**
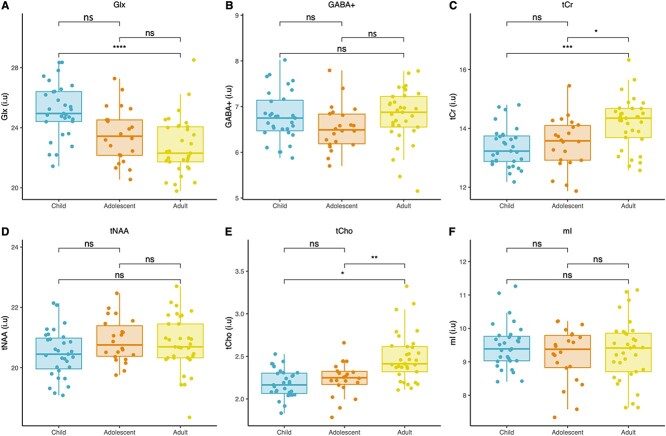
(A-F) estimated metabolite concentrations (i.u) per age group. Significant differences are indicated via connecting lines. Ns: Non-significant, ^*^P < 0.05, ^*^^*^P < 0.01, ^*^^*^^*^P < 0.001, ^*^^*^^*^^*^P < 0.0001.

Following ANCOVA’s, Tukey HSD post-hoc corrections were used to isolate specific differences in mean metabolite concentrations between age groups where appropriate. Results for estimated metabolite concentrations are shown in Fig. 3 (see Supplementary Fig. 2 for creatine-ratio data). Estimated concentrations of Glx significantly decreased from childhood to adulthood, while estimated concentrations of tCr and tCho significantly increased from childhood and adolescence to adulthood. For creatine ratio data, Glx/tCr, tNAA/tCr, mI/tCr and GABA+/tCr significantly decreased between age groups from childhood to adulthood, while tCho/tCr significantly increased from childhood to adulthood (Supplementary Fig. 2).

### Metabolite correlations with age per age group

Pearson correlation coefficients were calculated to assess the relationship between metabolite concentration and age *per age group.* In children, a significant negative correlation between estimated GABA+ concentrations and age (r = −0.5, *P* < 0.05) and a significant positive correlation between estimated tCr and tCho concentrations and age (r = 0.37, *P* < 0.05 and r = 0.36, *P* < 0.05 respectively) was observed. A significant negative correlation between estimated Glx and GABA+ concentrations and age was observed across children *and* adolescents (r = −0.61, *P* < 0.0001 and r = −0.32, *P* < 0.05 respectively). In adults, a significant negative correlation between estimated Glx concentrations and age was observed (r = −0.55, *P* < 0.001). For creatine-ratio data Glx/tCr significantly negatively correlated with age in children (r = −0.53, *P* < 0.05) and adults (r = −0.49, *P* < 0.05). GABA+/tCr significantly negatively correlated with age in children (r = −0.63, *P* < 0.05) and mI/tCr significantly negatively correlated with age in adults (r = −0.37, *P* < 0.05).

### Developmental modeling

**Fig. 4:**
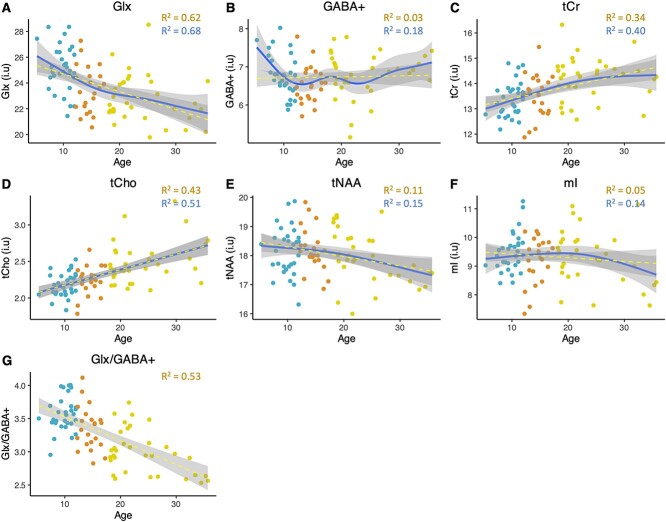
Linear (yellow) and GAM modeling (blue) of estimated metabolite concentrations (i.u) over the lifespan. R^2^ values for GAMs and linear regressions are shown. (G) Also shown is the relationship between estimated Glx/GABA+ ratio and age. Blue point = child, orange point = adolescent, yellow point = adult.

*Linear regression*: Linear models with metabolite concentration as the dependent variable and age as the independent variable and sex, IQ, SNR, FWHM, fGM and residuals of fit as predictors are shown in Fig. 4. Linear age effects were significant and negative for estimated concentrations of Glx (beta = −0.14, *P* < 0.05), and significant and positive for estimated concentrations of tCr (beta = 0.043, *P* < 0.05) and tCho (beta_sex-pooled_ = 0.020, p_sex-pooled_ = 0, beta_females_ = 0.023, p_females_ < 0.01, beta_males_ = 0.023, p_males_ < 0.01). No significant linear age effects were observed for estimated concentrations of tNAA, mI and GABA+.

For creatine ratio data linear age effects were significant and negative for tNAA/tCr (beta = −0.0063, *P* < 0.05) and Glx/tCr (beta = −0.013, *P* < 0.001), and significant and positive for tCho/tCr (sex pooled; beta_pooled_ = 0.001, p_pooled_ < 0.05; Supplementary Fig. 4).

Sex, FWHM, SNR and fGM were also added as predictors in a linear regression that tested for the effect of age on estimated Glx:GABA+ ratio. A significant and negative linear age effect was identified (beta = −0.03, *P* < 0.001; Fig. 4G). The same was observed for creatine-ratio Glx:GABA+ ratio (beta = −0.03, *P* < 0.001).

*General Additive Models:* GAMs are shown in Fig. 4. Significant non-linear effects of age were identified for estimated concentrations of tCr (edf = 1.43, *P* < 0.05), Glx (edf = 1.59, *P* < 0.001) and GABA+ (edf = 4.48, *P* < 0.05). Significant effects of age were strictly linear for tCho (edf = 1.00, *P* < 0.001). As to the shape of the GAMs, Fig. 4 shows that estimated concentrations (i.u) of Glx and GABA+ decline relatively sharply in childhood and adolescence. Glx concentrations then continue to gradually decrease across early adulthood. Concentrations of tCr (i.u) increase from childhood to early adulthood before tapering off, while tCho concentrations (i.u) increase linearly from childhood to early adulthood. Sex separated trajectories for tCho are found in Supplementary Fig. 5. Male and female trajectories are similar, with gradual increases in tCho concentrations across the lifespan, although this trend was more non-linear in males compared to females (edf_males_ = 2.14, *P* < 0.01; edf_females_ = 1, *P* > 0.05). For tNAA, when QM’s and fGM were controlled for, no significant (non-linear) effects of age were found.

Creatine-ratio concentrations show very similar trajectories to estimated concentrations for Glx, tCho and GABA+ (Supplementary Fig. 4). tNAA/tCr however shows a significant linear and negative association with age (edf = 1, *P* > 0.05).

### Metabolite interactions

**Fig. 5:**
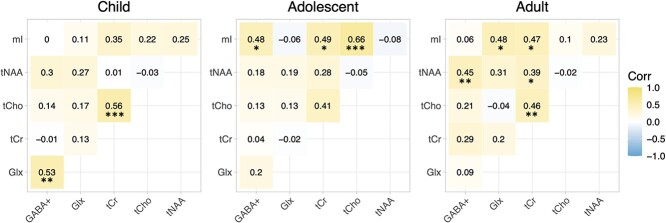
Correlation matrices for child, adolescent, and adult estimated metabolite concentrations (i.u). Significant Pearson correlation coefficients are shown ^*^P < 0.05, ^*^^*^P < 0.01, ^*^^*^^*^P < 0.001.

Correlations between individual neuro-metabolites differ between age groups (Fig. 5). In childhood only, GABA+ significantly positively correlates with Glx (r = 0.53, *P* < 0.001). In children and adults, we observe a significant positive correlation between tCho and tCr (r = 0.56; *P* < 0.001 and r = 0.46; *P* < 0.01 respectively). In adolescents and adults, we observe a significant positive correlation between tCr and mI (r = 0.49; *P* < 0.05 and r = 0.47; *P* < 0.05 respectively). In adolescents only we observe a significant positive correlation between GABA+ and mI (r = 0.48; *P* < 0.05) and tCho and mI (r = 0.66; *P* < 0.001). In adults only we observe a significant positive correlation between Glx and mI (r = 0.48; *P* < 0.05), GABA+ and tNAA (r = 0.45; *P* < 0.05) and tCr and tNAA (r = 0.39; *P* < 0.05).

### Associations with cognitive function

**Fig. 6:**
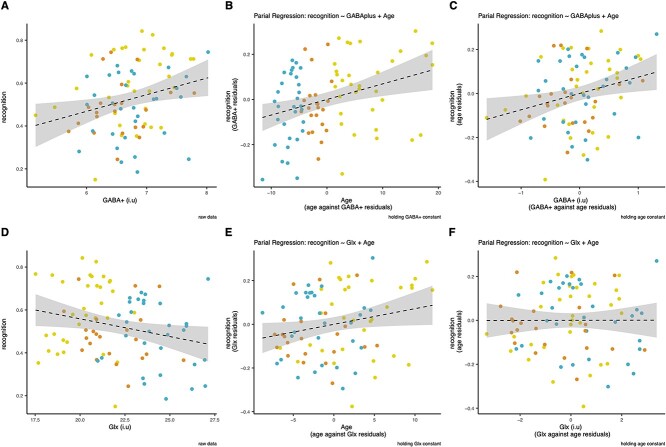
(A) GABA+ concentrations (i.u) plotted against recognition memory scores. (B) Partial regression plot of the linear regression: Recognition memory ~ GABA+ + age, with GABA+ held constant. (C) Partial regression plot of the linear regression: Recognition memory ~ age + GABA+, with age held constant. (D) Glx concentrations (i.u) plotted against recognition memory scores. (E) Partial regression plot of the linear regression: Recognition memory ~ Glx + age, with Glx held constant. (F) Partial regression plot of the linear regression: Recognition memory ~ age + Glx, with age held constant. Gray bars indicate standard error. Blue point = child, orange point = adolescent, yellow point = adult.

Estimated concentrations of GABA+ and Glx significantly correlated with recognition memory (r_GABA+_ = 0.27, p_GABA+_ < 0.05; r_Glx_ = −0.24, p_Glx_ < 0.05, Fig. 6). The linear regression model (recognition memory ~ age + GABA+ or Glx) was used to adjust for the effect of age. When age was held constant, GABA+ concentrations (i.u) showed a significant positive linear relationship with recognition memory scores (beta = 0.07, *P* < 0.05; Fig. 6C). For Glx (i.u), when age was adjusted for, we observed no significant relationship with recognition memory scores (beta = 0.00, *P* = 0.56; Fig. 6F).

## Discussion

### Developmental trajectories

We used edited ^1^H-MRS to determine the lifespan trajectory of several neurometabolites, and their association with individual differences in cognition, in a sample that spans development from childhood to early adulthood. Our primary data suggests that the trajectories of metabolite concentrations are not strictly linear, but rather asymptotic. For example, estimated Glx concentrations show a relatively sheep decrease in late childhood transitioning into early adolescence, before tapering off less steeply into early adulthood. Furthermore, we show that differences in cognitive function across development can be explained, in part, by differences in neurometabolism, specifically that of GABA+, the main inhibitory neurotransmitter. As such, the characterized developmental changes in neurometabolite concentrations are potentially mechanistically associated with cognitive development across the same period, and so may represent valid therapeutic targets during atypical neurodevelopment, where cognitive development is disrupted. Below, we briefly discuss the relevance of each metabolite and the limitations to our findings.

*Glx* Consistent with observations in the parietal lobe (Volk et al. 2019; Perdue et al. 2023), and in other brain regions (the basal ganglia: Ghisleni et al. 2015; occipital lobe: Shimizu et al. 2017; frontal: Perica et al. 2022), estimated PPC Glx concentrations decreased across relatively sharply mid-late childhood into adolescence. MRS Glx signal is largely localized to the glutamatergic presynaptic terminal (Posse et. 2007; Sailasuta et al. 2008). As such, these alterations in Glx are consistent with a developmental (post-natal) refinement of neural circuitry, whereby excitatory synapses, initially established in an excess, undergo activity dependent pruning (Huttenlocher and de Courten 1987; Zecevic et al. 1989; Huttenlocher 1990; Bourgeois et al. 1989; Zecevic and Rakic 1991; Bourgeois and Rakic 1993; Bourgeois et al. 1994; Huttenlocher and Dabholkar 1997; Geschwind and Rakic 2013; Selemon 2013). Functional outcomes of this structural refinement may contribute to previously observed increases in task induced activation of PPC regions during childhood, this having been associated with development of cognitive functions including arithmetic problem-solving abilities (Grabner et al. 2007; Chang et al. 2016). Changing neuronal and glial metabolic demands may also contribute to childhood declines in Glx, with cerebral glucose metabolism shown to increase from 3–4 years to 10 years of age (Chugani et al. 1987). An increasing metabolic rate is consistent with increasingly less free Glu (contributing to Glx signal) available across this period (Glu being an intermediate substrate in the mitochondrial Krebs cycle; Berl et al. 1961). Finally, decreases in neuronal/glial synthesis of Glu from Gln may also contribute, as studies in rats find that the cerebral expression of glutaminase, the enzyme that converts Gln to Glu, decreases from postnatal Day 14 through to adulthood (Boulland et al. 2003; Shimizu et al. 2017). The inherent limitations of our Gln-only estimates mean we are unable to scrutinize this using our Gln data.

In early adulthood the brain is reaching maturity (Giedd et al. 1999; Yakovlev and Lecours 1967; Lebel et al. 2008; Jernigan et al. 2011; Bethlehem et al. 2022), synaptic pruning is near completion (Huttenlocher 1979) and cerebral glucose metabolism rates decrease (Chugani et al. 1987). Consistent with this, we observe more gradual decreases in Glx concentrations. In older, adult only cohorts, researchers find a significant negative correlation between Glx/Glu concentrations and age in multiple brain regions including the parietal lobe (Gao et al. 2013, 20–76 years), the anterior cingulate cortex (Hädel et al. 2013, Glu, 19–55 years), the motor cortex (Kaiser et al. 2005, Glu, 24–68 years), the frontal lobe (Sailasuta et al. 2008, Glu, 21–71 years; Gao et al. 2013, Glx, 20–76 years; Marsman et al. 2013, Glu, 18–31 years), the basal ganglia and striatum (Sailasuta et al. 2008, Glu, 21–71 years; Ghisleni et al. 2015, Glu, 8–25 years). PPC Glx concentrations thus likely continue to decrease outside the scope of this study across mid/late adulthood. Of note, Sailasuta et al. 2008 (21–71 years) observed significant decreases in parietal Glx in adult men only, however, in a younger, larger cohort we observed no effect of sex on Glx trajectories across development.

*GABA+* Alike Glx, estimated GABA+ concentrations show a relatively sharp decline across childhood before plateauing in early adulthood (consistent with Ghisleni et al. 2015 and Perica et al. 2022). We should note that these results conflict with a recent meta-analysis by Porges et al. 2021 and findings by Saleh et al. 2020, which described an increase in frontal GABA+ concentrations during late childhood (8–12 years). These differences are likely explained by voxel placement, as biochemical development of the PPC differs markedly in timing to the frontal lobe, which is one of the last brain regions to mature (Ouyang et al. 2019). Increases in GABA+ concentrations (thought to reflect myelination and increased synaptic activity; Porges et al. 2021) may thus occur earlier than the scope of this study in PPC. Similar increases in Glx may also precede our trajectories, as Perdue et al. 2023 observed that temporal–parietal Glx concentrations increased between 2 and 6 years of age before decreasing from mid childhood, consistent with our observations in the PPC.

GABAergic activity at GABA_A_ ionotropic receptors (GABA_A_R), widely expressed across the central nervous system, mediates fast inhibitory signaling in the mature adult brain by extra-synaptic and synaptic membrane hyperpolarization (Birnir et al. 2000; Vithlani et al. 2011; Brickley and Mody 2012). For a period of time in the immature brain, GABA activity at GABA_A_R is however excitatory (depolarizing), with the switch from excitatory to inhibitory GABA_A_R mediated neuronal responses thought to occur during the post-natal period (Rivera et al. 1999; Dzhala et al. 2005; Ghit et al. 2021) and concurrent with developmental changes in GABA_A_R currents and subunits (Fritschy et al. 1994; Taketo and Yoshioka 2000; Okada et al. 2000; Pandit et al. 2017). Childhood decreases in GABA+ concentration, synchronous with Glx declines, likely reflects this post-natal GABAergic maturation, and the establishment of a homeostatic balance between the emerging inhibitory activity of GABA and the excitatory activity of glutamate. Disruptions to the establishment of this balance are thought to contribute to neurodevelopmental disorders such as autism (Rubenstein and Merzenich 2003), epilepsy (Symonds 1959), and schizophrenia (Gao and Penzes 2015).

In neuro-typical individuals, we observe the Glx:GABA+ ratio to significantly decrease from childhood to early adulthood, driven largely by declines in Glx concentrations. This is consistent with a developmental shift in the balance of neural activity towards inhibition (Zhang et al. 2011; Ghisleni et al. 2015; Baumer et al. 2020) and work in mice finding a developmental depression of cortical excitatory activity (amplitude of excitatory post synaptic potentials and synaptic release probability) but not inhibitory activity (Reyes and Sakmann 1999; Oswald and Reyes 2008; Feldmeyer and Radnikow 2009). Increased neuronal excitability in early development facilitates a critical period of synaptic plasticity, essential for the establishment and consolidation of neural circuitry, before increasing inhibitory activity with local circuit refinement to increase signal to noise and so sensitivity (Citri and Malenka 2008; Zhang et al. 2011).

*tCho* Age-associated increases in tCho concentrations have been observed in several brain regions in adult only cohorts (Chang et al. 1996, 19–78 years; Lind et al. 2020, ACC, 18–79 years; Ding et al. 2016, WM, 20–70 years; Schmitz et al. 2018, 22–73 years; Pfefferbaum et al. 1991, frontal lobe, 25–73 years; Sailasuta et al. 2008, frontal WM, 21–73 years). We observe this relationship to be true in our developmental cohort spanning from mid childhood to early adulthood. While MRS tCho signal has contributions from phosphatidylcholine (a phospholipid highly abundant in the cell membrane; Michel et al. 2006) and glycerophosphocholine (a cellular osmolyte), it has been demonstrated that free choline molecules principally contribute to the detected MRS signal (Miller et al. 1996). Our results may thus reflect the breakdown of neural/glial membrane across this period (less choline sequestered as phosphatidylcholine), consistent with a developmental “cortical thinning,” by which cortical thickness decreases across childhood and adolescence due to the refinement of structural connectivity in GM (Giedd et al. 1999; Sowell et al. 2004; Asato et al. 2010; Ducharme et al. 2016; Tamnes et al. 2017; Fuhrmann et al. 2022). Such parietal cortical thinning has been previously associated with cognitive development across adolescence (Sowell et al. 2002; Sowell et al. 2004; Shaw et al. 2006). As for the observed sex differences in tCho trajectories (Bohacek et al. 2008; Hädel et al. 2013), the potentiating effects of estrogen on choline acetyltransferase expression, an enzyme responsible for tCho conversion into acetylcholine, may contribute (Bohacek et al. 2008; Hädel et al. 2013), and also explain why we observe sex differences in tCho concentrations only *after* puberty.

*tCr* Age-associated increases in tCr concentrations have been previously observed across various age ranges; Marsman et al. 2013 (medial frontal cortex, 18–31 years), Schmitz et al. 2018 (whole brain MRSI, 30–67 years), Blüml et al. 2013 (cerebral GM & parietal lobe, 5 months—12 years), Reyngoudt et al. 2012 (posterior cingulate cortex, 18–76 years), Perdue et al. 2023 (left temporo-parietal lobe, 2–11 years) and Kaiser et al. 2005 (motor cortex and corona radiata, 24–68 years). We find however that PPC tCr increases are steeper during childhood and adolescence compared to early adulthood. The creatine-phosphocreatine flux regulates ATP storage and fast release within neurons, and as such results for MRS tCr concentrations (composed of free creatine and phosphocreatine) are likely reflective of developmental changes in neuronal activity (Wallimann et al. 1992; Rae 2014). For example, increasing tCr concentrations across childhood and adolescence is consistent with the previously discussed childhood increases in cerebral metabolic activity (Chugani et al. 1987), but also increasing functional connectivity of posterior parietal regions with temporal, frontal and visual regions across the same period, associated with development of reading ability (phonological short term memory), episodic memory, visual perception and working memory (Edin et al. 2007; Fair et al. 2008; Dosenbach et al. 2010; Chang et al. 2016; O’Rawe et al. 2019; Kwon et al. 2002; Ghetti and Bunge 2012; Demaster and Ghetti 2013; Lee et al. 2016, Vinette and Bray 2015). By early adulthood brain development slows, and as such neuronal energy demands begin to plateau (Chugani et al. 1987; Bethlehem et al. 2022), reflected in the plateauing of tCr trajectories.

This instability of (water scaled, tissue corrected) creatine concentrations across the developmental stage studied likely drives the differences in estimated and creatine ratio metabolite trajectories, with creatine-ratio trajectories likely failing to reflect real changes in tissue physiology. This is compelling evidence opposing the exclusive use of creatine-ratio MRS data for case–control/cross sectional study designs, a practice that persists, and we consider this an important outcome of our study. Our interpretation of developmental metabolite trajectories (above and below) focuses on water scaled, tissue corrected, estimated metabolite concentrations only.

*tNAA and mI* After controlling for several confounding factors, we find that PPC tNAA and mI concentrations show no association with age across development. While for mI this is consistent with previous work (Blüml et al. 2013; Perdue et al. 2023), these findings are inconsistent with work in a larger child cohort finding that temporo-parietal estimated tNAA concentrations increase across childhood. NAA is highly concentrated within the neuronal cell body (Rae 2014), but also within oligodendrocytes where it has also been shown to have roles in maintaining myelin sheath integrity (Singhal et al. 2017). As such, tNAA increases have been suggested to reflect developmental myelination and axon extension (temporo-parietal region; Perdue et al. 2023; 2–11 years). Differences in results may be explained by our more limited sample size, our controlling for differences in MRS data quality, but also regional differences in myelination, with myelination in temporal regions shown to succeed myelination in parietal regions (Deoni et al. 2011). Thus, myelination mediated increases in PPC tNAA may no longer be discernible by mid childhood. Studies in older, adult only cohorts find age related declines in tNAA/NAA concentrations (Saleh et al. 2020, occipital lobe, 18–87 years; Kirov et al. 2008, 20–90 years, whole brain MRSI; Ding et al. 2016, Schmitz et al. 2018, 20–70 years, whole brain MRSI; Kasier et al. 2005, 24–68 years, motor cortex). As NAA is synthesized in the neural/glial mitochondria, tNAA declines in adult only populations may reflect a slow and increasing mitochondrial impairment due to the gradual accumulation of oxidative stress (Patel and Clark 1979; Demougeot et al. 2001; Zuendorf et al. 2003; De Moura et al. 2010; Chistiakov et al. 2014; Rae 2014; Siddiqui et al. 2016). Furthermore, we have previously discussed evidence of a developmental depression of cortical excitatory activity, and so decreases in NAAG concentrations, a precursor to glutamate but also a negative feedback modulator of glutamatergic synaptic activity (Moffett et al. 2007; Nguyen et al. 2019; Morland and Nordengen 2022), may also contribute to adulthood tNAA declines. These observations are beyond the scope of the developmental age range studied here.

In summary, the characterized metabolite trajectories are likely underpinned by the developmental refinement, maturation, and myelination of excitatory and inhibitory brain circuitry in the postnatal period. Trajectories diverge due to the differing developmental process(s) governing each metabolite.

### Metabolite-metabolite interactions

Metabolite correlations appear stronger during early adulthood compared to childhood, likely due to the maturation of neuronal/glial metabolic pathways across this period. For example, variations in Glu-GABA balance across development (Reyes and Sakmann 1999; Oswald and Reyes 2008; Feldmeyer and Radnikow 2009; Zhang et al. 2011), potentially linked to post-natal alterations in the expression of enzymes involved in Glu metabolism (Hertz 2013), likely explain the uncoupling of Glx and GABA+ concentrations from childhood to adulthood (as excitatory activity is depressed) and the variable association between tNAA and GABA+ across the different age groups (NAA and NAAG precursors for glutamate production and glutamate being a precursor for GABA; Mathews and Diamond 2003; Bak et al. 2006; Wang et al. 2007; Nguyen et al. 2019).

Similarly, dynamic developmental processes likely explain why in children and adolescents’ energy production (tCr/tNAA) and membrane integrity (tCho) markers are uncoupled. High rates of developmental synaptic pruning during this period (Huttenlocher 1979; Bourgeois and Rakic 1993) mean comprises in membrane integrity are concurrent with increases in neural energy demands (Huttenlocher 1979; Chugani et al. 1987). In the adult brain, however, tCr and tNAA, and tCr and tCho positively associate (consistent with Rodriguez-Nieto et al. 2023), reflective of the increasingly stable neuronal energy demands and/or neural integrity (less synaptic pruning; Huttenlocher 1979; Wallimann et al. 1992; Rae 2014; Siddiqui et al. 2016; Rodriguez-Nieto et al. 2023 ).

Finally, mI concentrations positively associate with several metabolites, including tCr, in adolescents and adults, consistent with work finding that oral administration of tCr increases cerebral mI concentrations, a response thought to regulate of cell osmolarity (Brand et al. 1993; Strange et al. 1993; Michaelis et al. 1999; Su et al. 2023). As osmotic forces have been shown to contribute to neuronal differentiation (Kourouklis et al. 2023), such homeostatic regulation of cell osmolarity maybe altered the early developing brain, potentially explaining why this association is absent in children.

### Memory correlates

Across all age groups PPC GABA+ concentrations significantly positively associated with recognition memory scores. We did not see any significant relationship between PPC Glx concentrations and memory scores (when age was accounted for), supporting the specificity of this association.

PPC GABA may contribute to recognition memory performance via the tuning of pyramidal neuron synchronization within the hippocampal-parietal network. The PPC, acting within the hippocampal-parietal memory network, has been increasingly implicated in episodic memory (including recognition; Donaldson and Rugg 1998; Wagner et al. 2005; Cabeza et al. 2008; Vincent et al. 2006; Ciaramelli et al. 2008; Wang et al. 2010; Riggins et al. 2016; Ngo et al. 2017; Fan et al. 2021). Within this hippocampal-parietal network, theta (low frequency) and gamma (high frequency) oscillations of pyramidal neuron activity has been associated with episodic memory performance (Nyhus and Curran 2010; Uhlhaas et al. 2010; Heusser et al. 2016; Natu et al. 2019; Griffiths et al. 2021). GABA has been shown to regulate this oscillatory activity (Cobb et al. 1995; Whittington et al. 1995; Traub et al. 1996; Chen et al. 2014; Dubey et al. 2022). This evidence thus provides a link between PPC GABA+ concentrations and recognition memory performance.

### Limitations

We note several limitations to our study. First, metabolite concentrations across all ages in our study population were post-hoc corrected using literature derived adult tissue water and metabolite T1 and T2 relaxations values (Gasparovic et al. 2006; Oeltzschner et al. 2020; Hui et al. 2022). Due to changes in brain tissue composition and particularly water content, these values likely vary in children (Christiansen et al. 1993; Kirov et al. 2008). We do however report very similar trajectories for creatine-referenced metabolite data, for which no post hoc corrections (T1/T2/tissue) were made, and as such it is unlikely that the trends identified in water referenced data are artifacts of this post-hoc correction.

Macromolecule (MM) contribution to the GABA+ signal was also post-hoc corrected using existing MM basis set functions modeled on data from adult participants (Oeltzschner et al. 2020; Hui et al. 2022). Thus, while Hui et al. (2022) found no significant age associated changes in MM signal, it cannot be ruled out that childhood differences in MM contribution to GABA+ does not contribute to the observed GABA+ trajectory (Bell et al. 2021). Macromolecule supressed editing can overcome this limitation, however it is highly motion sensitive and achieves a significantly lower SNR compared to MM unsuppressed MEGA-PRESS (Mikkelsen et al. 2017; Bell et al. 2021). In the future, characterization of metabolite and tissue water T1/T2 relaxation and MM signal contribution across the lifespan (and for specific brain regions) will enable more tailored corrections of metabolite concentrations better addressing these accuracy concerns.

Beyond T1 and T2 relaxation and MM contributions, metabolite basis sets are also tailored towards adult data potentially explaining the increased fitting errors found in the child cohort. However, we note that fitting errors have low variability and are still within normative ranges (Mikkelsen et al. 2017) and it is unlikely that this has broadly affected our results. We additionally used fit residuals as a covariate in our linear and non-linear models, with age still significantly predicting metabolite concentrations and unchanged levels of significance.

Similarly, within our cohort, we observed IQ scores to be significantly greater in adults compared to children and adolescents. As such, IQ was also included as a covariate in our linear and non-linear models, with age still significantly predicting metabolite concentrations. We also identified no significant effect of IQ on metabolite concentrations, when added to ANCOVA’s, linear regression and non-linear models. Thus, we are confident that this has not had a broad effect on our results overall.

Finally, as a cross-sectional study with a limited sample size, individual variation is likely to have contributed to theorized trajectories, especially past 30 years of age where the data was sparser and so non-linear models became more heavily influenced by individual data points. Thus, while adjusted R^2^ values obtained from GAM models were greater than adjusted R^2^ values obtained by linear regression for all metabolites (Fig. 5), this may partially stem from the nature of the fitting method. In fact, we report linear regression models to prevent overinterpretation of non-linear data trends that maybe artifacts from GAM overfitting, however, although to a lesser degree, linear regressions are still limited by the sample size. In future, larger, longitudinal datasets must be analyzed, increasing power and reliability of identified data trends.

## Conclusion

We examined the lifespan trajectories of six essential MRS metabolites measured from a PPC voxel at a single site. Glx and GABA+ concentrations exhibited a steep decline across late childhood into adolescence, reflecting early circuit refinement and the developmental fine-tuning of the balance between excitatory and inhibitory neuronal activity. tCr concentrations increased during childhood and adolescence, aligning with increasing neural energy demands, before tapering off into early adulthood. Meanwhile, tCho concentrations showed a linear increase across the developmental period studied, likely reflective of PPC structural circuit refinement. MI and tNAA concentrations remained relatively stable. The characterized trajectories were associated with cognitive outcomes, as GABA+ concentrations demonstrated a significant positive association with recognition memory scores. This association potentially reflects the role of PPC GABAergic activity in modulating neuronal oscillatory activity within the developing hippocampal-parietal memory network.

## Supplementary Material

supplementary_matierals_revised_bhae046

## Data Availability

Data are available through: https://osf.io/uv67s/?view_only=2896964f249f4249bbfdeccb0b96bb73. Osprey 2.4.0 is available through: https://github.com/schorschinho/osprey.
