# Supplementary material for: The developmental trajectory of 1H-MRS brain metabolites from childhood to adulthood
Source: Cereb Cortex. 2024 Mar 1;34(3):bhae046. doi: 10.1093/cercor/bhae046 (PMC10908220; doi:10.1093/cercor/bhae046)
Supplement: supplementary_matierals_revised_bhae046 [file supplementary_matierals_revised_bhae046.docx]

Supplementary table 1**.** Mean (SD) estimated (i.u) and creatine-ratio (/tCr) metabolite concentrations for each age group.

| Metabolite | Child | Adolescent | Adult | p value |
| --- | --- | --- | --- | --- |
| tCr (i.u) off | 13.358 (0.675) | 13.500 (0.858) | 14.181 (0.857) | 7.07e-05 *** |
| tCr/tCr off | - | - | - | - |
| tNAA (i.u) off | 20.473 (0.784) | 20.901 (0.719) | 20.797 (0.915) | 0.11354 |
| tNAA/tCr off | 1.767 (0.112) | 1.786 (0.113) | 1.690 (0.099) | 0.000524 *** |
| tCho (i.u) off | 2.180 (0.163) | 2.236 (0.195) | 2.489 (0.287) | 4.53e-06 *** |
| tCho/tCr off | 0.202 (0.013) | 0.205 (0.017) | 0.217 (0.022) | 0.00294 ** |
| Glx (i.u) diff | 25.148 (1.782) | 23.522 (1.804) | 22.753 (1.878) | 0.00176 ** |
| Glx/tCr diff | 1.809 (0.156) | 1.676 (0.159) | 1.544 (0.149) | 0.000103 *** |
| Glx (i.u) off | 24.163 (1.647) | 21.856 (1.685) | 20.212 (1.415) | 5.12e-07 *** |
| Glx/tCr off | 1.532 (0.145) | 1.377 (0.147) | 1.178 (0.102) | 1.79e-11 *** |
| Glu (i.u) diff | 23.860 (1.877) | 22.038 (1.710) | 21.186 (1.405) | 2.50e-05 *** |
| Glu/tcr diff | 1.684 (0.159) | 1.539 (0.126) | 1.409 (0.110) | 1.05e-08 *** |
| Glu (i.u) off | 21.699 (1.666) | 19.672 (1.397) | 17.711 (1.269) | 4.34e-09 *** |
| Glu/tcr off | 1.738 (0.139) | 1.559 (0.162) | 1.371 (0.112) | 1.88e-12 *** |
| Gln (i.u) diff | 1.714 (0.822) | 1.872 (0.999) | 1.937 (1.195) | 0.6346 |
| Gln/tCr diff | 0.125 (0.060) | 0.137 (0.077) | 0.134 (0.085) | 0.787 |
| Gln (i.u) off | 2.830 (0.701) | 2.515 (0.604) | 2.791 (0.645) | 0.160 |
| Gln/tCr off | 0.206 (0.048) | 0.181 (0.042) | 0.192 (0.044) | 0.251 |
| GABA+ (i.u) diff | 6.787 (0.542) | 6.539 (0.489) | 6.794 (0.594) | 0.159 |
| GABA+/tCr diff | 0.426 (0.038) | 0.408 (0.037) | 0.394 (0.034) | 0.000731 *** |
| mI (i.u) off | 0.009 (0.014) | 0.010 (0.012) | 0.006 (0.010) | 0.95315 |
| mI /tCr off | 0.841 (0.060) | 0.807 (0.064) | 0.778 (0.070) | 0.02567 * |

Note: 31 children, 22 adolescents and 33 adults. Off = quantified from edit-off spectrum, diff = quantified from difference spectrum. p value indicates significance from multiple comparison ANCOVA’s to test main effect of age group on metabolite concentrations (IQ, sex and residuals of fit as covariates). *p<0.05, **p<0.005, ***p<0.001, ****p<0.0001.

Supplementary table 2. p values of intercepts obtained from multiple comparison ANCOVA’s with estimated (i.u) or creatine ratio (/tCr) metabolite concentration as the dependant variable and IQ, sex, fit residuals, FWHM, fGM, SNR and age group as independent variables.

| Metabolite | | Estimated concentration (i.u) | | | | | | | | Creatine scaled concentration (/tCr) | | | | | |  | |
| --- | --- | --- | --- | --- | --- | --- | --- | --- | --- | --- | --- | --- | --- | --- | --- | --- | --- |
|  | IQ | | sex | fit residual | age group | SNR | FWHM | fGM | IQ | | Sex | fit residual | age group | SNR | FWHM | | fGM |
| tCr off | 0.161751 | | 0.523849 | 0.082703 | 7.07e-05 *** | 0.000667 *** | 0.000495 *** | 0.185970 | NA | | NA | NA | NA | NA | NA | | NA |
| tNAA off | 0.05405 | | 0.08802 | 0.73070 | 0.11354 | 0.27338 | 0.00634 ** | 0.16840 | 0.841506 | | 0.084316 | 0.177962 | 0.000524 *** | 0.014082 * | 0.001832 ** | | 0.145580 |
| tCho off | 0.517191 | | 0.001819 ** | 0.000294 *** | 4.53e-06 *** | 0.007270 ** | 0.181530 | 0.037280 * | 0.12867 | | 0.38759 | 0.24757 | 0.00294 ** | 0.94141 | 0.88670 | | 0.24757 |
| Glx diff | 0.44420 | | 0.32019 | 7.01e-06 *** | 0.00176 ** | 0.40525 | 0.52990 | 0.90713 | 0.170564 | | 0.943165 | 6.13e-06 *** | 0.000103 *** | 0.280965 | 0.973818 | | 0.534437 |
| Glx off | 0.0693 | | 0.7641 | 3.27e-15 *** | 5.12e-07 *** | 0.4228 | 0.0637 | 0.0785 | 0.0198 * | | 0.7990 | 1.89e-15 *** | 1.79e-11 *** | 0.0902 | 4.48e-07 *** | | 0.1688 |
| Glu diff | 0.4577 | | 0.7267 | 7.21e-08 *** | 2.50e-05 *** | 0.5701 | 0.0903 | 0.2216 | 0.191 | | 0.454 | 1.51e-07 *** | 1.05e-08 *** | 0.149 | 0.139 | | 0.101 |
| Glu off | 0.09004 | | 0.75472 | < 2e-16 *** | 4.34e-09 *** | 0.36062 | 0.04173 * | 0.00293 ** | 0.0428 * | | 0.4419 | 2.37e-16 *** | 1.88e-12 *** | 0.1327 | 5.44e-08 *** | | 0.0319 * |
| Gln off | 0.815 | | 0.0226 * | 0.5882 | 0.6346 | 0.5418 | 0.1361 | 0.068 | 0.725 | | 0.027 * | 0.719 | 0.787 | 0.840 | 0.190 | | 0.137 |
| Gln off | 0.421 | | 0.217 | 0.698 | 0.160 | 0.978 | 0.821 | 0.068 . | 0.233 | | 0.251 | 0.427 | 0.251 | 0.202 | 0.897 | | 0.116 |
| GABA+ diff | 0.6751 | | 0.9656 | 0.3093 | 0.1593 | 0.5051 | 0.1329 | 0.0768 | 0.484856 | | 0.787154 | 0.869537 | 0.000731 *** | 0.184080 | 0.015757 * | | 0.062988 |
| mI off | 0.49613 | | 0.95315 | 0.10148 | 0.70942 | 0.00281 ** | 0.79591 | 0.74466 | 0.93383 | | 0.59706 | 0.00649 ** | 0.02567 * | 0.28083 | 0.26676 | | 0.86093 |

Supplementary table 3. Estimated (i.u) and creatine ratio (tCr) concentrations of tCho per age group per sex.

| Metabolite | Child | | | | Adolescent | | Adult | |
| --- | --- | --- | --- | --- | --- | --- | --- | --- |
|  | | F | M | F | | M | F | M |
| tCho (i.u) | | 2.13 (0.12) | 2.23 (0.19) | 2.20 (0.19) | | 2.27 (0.20) | 2.35 (0.17) | 2.60 (0.32) |
| tCho/tCr | | 0.20 (0.010) | 0.20 (0.015) | 0.20 (0.019) | | 0.21 (0.016) | 0.20 (0.015) | 0.23 (0.021) |

Note: F = female, M = male.

Supplementary table 4. p values obtained from Wilcoxon Rank test for differences in estimated (i.u) and creatine ratio (/tCr) tCho concentrations between sexes for each age group.

| Metabolite | Child | Adolescent | Adult | All |
| --- | --- | --- | --- | --- |
| tCho (i.u) | P = 0.0784 | P = 0.381 | P = 0.0106* | 0.0059 |
| tCho/tCr | P = 0.32 | P = 0.67 | P = 0.0021* | 0.0056 |

Note: * indicates significance after Bonferroni multiple comparison correction.

Supplementary figure 1. (A-H) Tukey HSD post-hoc correction was used to isolate significant differences in estimated metabolite concentrations (i.u) between age groups. Significant differences are indicated via connecting lines. ns: non-significant, *p < 0.05, **p < 0.01, ***p < 0.001, ****p < 0.0001. tCr, tNAA, tCr, Glx, Gln, Glu and mI were quantified from the edit-OFF spectrum, while GABA+ was quantified from the difference spectrum.

Supplementary figure 2. (A-G) Tukey post-hoc correction was used to isolate significant differences in creatine-ratio metabolite concentrations (i.u) between age groups. Significant differences are indicated via connecting lines. ns: non-significant, *p < 0.05, **p < 0.01, ***p < 0.001, ****p < 0.0001. tCr, tNAA, tCr, Glx, Gln, Glu and mI were quantified from the edit-OFF spectrum, while GABA+ was quantified from the difference spectrum.


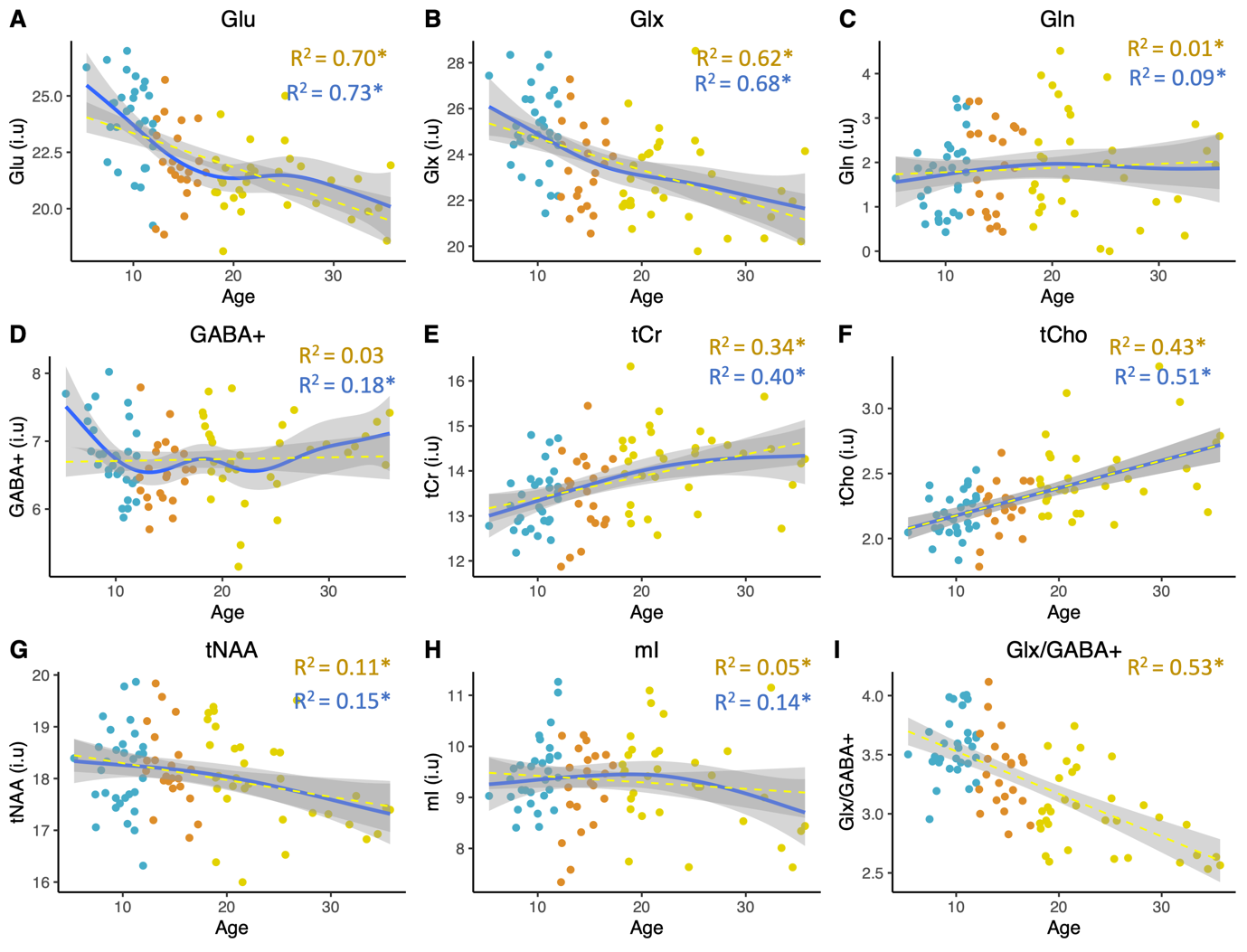


Supplementary figure 3. Linear (yellow) and GAM modelling (blue) of metabolite (A-H) estimated concentrations (i.u) over the lifespan. R^2^ values (and significance of the model) for GAMS and linear regression models are shown. (I) Also shown is the relationship between Glx/GABA+ ratios and age (linear regression). Blue point = child, orange point = adolescent, yellow point = adult, * p < 0.05.

Supplementary figure 4. Linear (yellow) and GAM modelling (blue) of metabolite (A-H) creatine ratio concentrations over the lifespan. R^2^ values (and significance of the model) for GAMS and linear regression models are shown. (I) Also shown is the relationship between Glx/GABA+ ratios and age (linear regression). Blue point = child, orange point = adolescent, yellow point = adult, * p < 0.05.

**

Supplementary figure 5. Linear (yellow) and non-linear GAM modelling (blue) of (A-D) estimated (i.u) and (E-H) creatine ratio (/tCr) metabolite concentrations of Gln and tCho across the lifespan when data was grouped by sex.


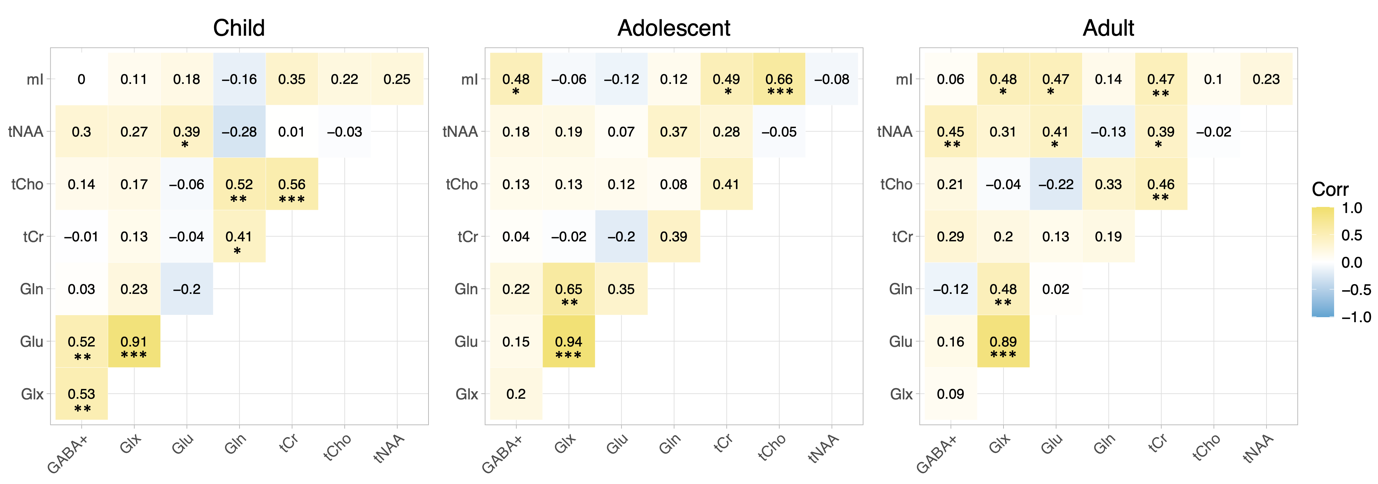


Supplementary figure 6. Correlation matrices for child, adolescent and adult estimated metabolite concentrations, with the Pearson correlation coefficients (r) shown. Yellow = positive correlation, blue = negative correlation and white = no correlation. Significant correlations are shown *p<0.05, **p<0.005, ***p<0.001, ****p<0.0001.

Supplementary table 5. Pearson correlation coefficients for estimated metabolite concentration and recognition memory scores.

| Metabolite | Pearsons r | p value |
| --- | --- | --- |
| Glu: recognition | *-0.11* | *0.311* |
| Glx: recognition | *-0.1* | *0.34* |
| GABA+: recognition | *0.27* | *0.0126** |
| Age: recognition | *0.32* | *0.0026** |

*Note:* *p<0.05, **p<0.005, ***p<0.001, ****p<0.0001.
